# Influence of storage temperature and low‐temperature conditioning on the levels of health‐promoting compounds in Rio Red grapefruit

**DOI:** 10.1002/fsn3.429

**Published:** 2016-10-02

**Authors:** Priyanka R. Chaudhary, Xiang Yu, Guddadarangavvanahally K. Jayaprakasha, Bhimanagouda S. Patil

**Affiliations:** ^1^ Vegetable and Fruit Improvement CenterDepartment of Horticultural SciencesTexas A&M UniversityCollege StationTX 77845USA

**Keywords:** Chilling injury, grapefruit, health‐promoting compounds, low‐temperature conditioning, quarantine

## Abstract

Commercial operations use low‐temperature conditioning of citrus fruits to reduce the incidence of chilling injury (CI) during cold storage and quarantine treatments. Rio Red grapefruits (*Citrus paradisi* Macf) were stored for 12 weeks at 11°C or 5°C; an additional set was temperature conditioned at 16°C for 7 days before storing at 5°C (CD). Every 3 weeks, samples were assessed for chilling injury (CI) and health‐promoting compounds such as ascorbic acid, carotenoids, limonoids, flavonoids, and furocoumarins. Low‐temperature conditioning significantly reduced CI but did not affect the total soluble solids, acidity, and ripening ratio. After 12 weeks of storage, grapefruits showed no significant differences in lycopene, narirutin, poncirin, furocoumarins, and radical scavenging activity in all the three treatments. Limonin was significantly higher (*p *<* *.05) in CD fruits, nomilin was significantly higher in fruits stored at 11°C, whereas fruits stored at 5°C had lower levels of naringin, neohesperidin, and didymin after 12 weeks of storage. Low‐temperature conditioning treatment helped fruits to retain similar or higher levels of most of the health‐promoting compounds by the end of storage period while maintaining better quality than the nonconditioned fruits.

## 1 Introduction

The Mexican fruit fly (*Anastrepha ludens,* Loew) is a quarantine pest of citrus, and lays eggs inside the fruit. The larvae feed on the fruit flesh and their presence limits marketing and export of the citrus fruits. To be exported, fruits must meet phytosanitary requirements and undergo quarantine treatments such as fumigation with methyl bromide (Hallman & Thomas, 2011), heat treatment (Armstrong & Mangan, 2007), cold treatment (Hill, Rigney, & Sproul, 1988), or irradiation (Hallman, 1999). However, methyl bromide is a class I ozone‐depleting chemical, indicating the need for alternative techniques. Low‐temperature quarantine treatment, where fruits are stored at a temperature below the thermal tolerance of the pests, provides a good alternative. Temperature also affects respiration and other metabolic activities in fruits and vegetables, possibly affecting their quality and shelf‐life.

Grapefruit must be stored below 5°C for disinfestation; however, certain tropical and subtropical fruits, including grapefruit, develop chilling injury (CI) when stored at low temperature. Various factors affect the incidence of CI in citrus fruits, such as variety, harvest time, fruit maturity, temperature, and waxing (Brown, Petracek, Chambers, Dou, & Pao, 1998). For example, a previous study reported that an increase in temperature during the winter increased the chilling tolerance in grapefruits after harvest (Kawada, Grierson, & Soule, 1979). Also, fruits harvested from the exterior of the canopy are more susceptible to CI (Purvis, 1984). The main factor affecting CI of grapefruit is moisture loss from the fruits (Purvis, 1984) and, therefore, treatments that reduce moisture loss, such as waxing (Forney & Lipton, 1990), can reduce the incidence of CI.

Temperature conditioning before the fruits are stored at low temperature can also prevent CI. The influence of conditioning treatment on citrus peels and fruit quality has been studied extensively (Gonzalez‐Aguilar, Zacarias, Mulas, & Lafuente, 1997; Maul, McCollum, Guy, & Porat, 2011; Tietel, Weiss, Lewinsohn, Fallik, & Porat, 2010). Porat et al. reported conditioning grapefruit at 16°C for 7 days before cold storage effectively minimizes chilling injury (Maul, McCollum, Popp, Guy, & Porat, 2008; Porat, Pavoncello, Peretz, Ben‐Yehoshua, & Lurie, 2000). Several studies on low‐temperature conditioning treatment effect on molecular mechanisms involved in chilling tolerance have been conducted (Maul et al., 2008; Sapitnitskaya et al., 2006). However, the effect of low‐temperature conditioning on Rio Red grapefruit phytochemicals present in juice vesicles has not been investigated. The phytochemicals/health‐promoting compounds predominantly present in grapefruit are ascorbic acid, carotenoids, limonoids, flavonoids, and furocoumarins; dietary intake of these compounds reportedly reduces the risks of certain chronic diseases (Patil, Brodbelt, Miller, & Turner, 2006). Therefore, maintaining the levels of these health‐promoting compounds during postharvest storage has important implications for human health. Studies on the effect of temperature and storage period on the health‐promoting compounds in the juice vesicles, as well as on the maintenance of quality of grapefruit will provide key information for maintaining phytochemical contents. This study investigated the influence of cold storage and low‐temperature conditioning on levels of health‐promoting compounds in Rio Red grapefruit, examining the levels of ascorbic acid, carotenoids, limonoids, flavonoids, furocoumarins, total phenolics, and radical scavenging activity, during a 12‐week storage period.

## 2 Materials and Methods

### 2.1 Plant material

Rio Red grapefruits of uniform size were harvested on February 10, 2010, from three different blocks (250 fruits from each block) from Rio Queen Citrus, a commercial grove in the Rio Grande Valley in South Texas.

### 2.2 Chemicals

Sodium hydroxide, L‐ascorbic acid, butylated hydroxytoluene (BHT), lycopene, β‐carotene, narirutin, naringin, neohesperidin, didymin, poncirin, limonin, 6′, 7′‐dihydroxybergamottin (DHB), catechin, Folin‐Ciocalteu reagent, sodium carbonate, and 2,2‐diphenyl‐1‐picrylhydrazyl (DPPH) were procured from Sigma Aldrich Co. (St. Louis, MO, USA). Analytical grade solvents were obtained from Fisher Scientific Research (Pittsburgh, PA, USA).

### 2.3 Treatment and storage

Approximately 250 grapefruits were harvested from each block (replication) and were further divided into three lots of 80 fruits for each temperature treatment, 11°C, 5°C, and conditioning treatment (CD) in which fruits were conditioned at 16°C for 7 days and then stored at 5°C. Fruits in all the three treatments were stored for 12 weeks at 90% relative humidity. Three juice subsamples were collected at an interval of 3 weeks from each replication. Each juice sample (subsamples) was prepared by blending three peeled grapefruits and were stored at −80°C until further analysis (*n* = 9 per treatment, 3 replications × 3 subsamples).

### 2.4 Total soluble solids and total acidity

Total soluble solids (TSS) were measured using a hand refractometer (American Optical Corp., South Bridge, MA, USA) and results were expressed as Brix. A DL 22 Food and Beverage analyzer (Mettler Toledo, Columbus, OH, USA) was used to measure the total acidity of juice. Grapefruit juice (5 ml) was mixed with 50 ml of nanopure water and titrated with 0.1‐N sodium hydroxide, and total acidity was expressed as percent citric acid. Ripening ratio was calculated as the ratio of TSS to total acidity (TSS/total acidity). Each treatment had three replications containing two samples each (*n* = 6).

### 2.5 Chilling injury index

Chilling injury was evaluated and expressed as CI index (Porat et al., 2000). Grapefruits were sorted into four groups based on their severity of CI: score 0 (no pitting), score 1 (a few scattered pits), score 2 (pitting covering up to 30% of the fruit surface), and score 3 (extensive pitting covering more than 30% of the fruit surface). The CI index for each treatment was further calculated by multiplying the number of fruits in each category by their score and dividing the sum of each treatment by the total number of fruits assessed. All treatments included three replications, each replication containing 10 fruits (*n* = 30).

### 2.6 Ascorbic acid determination

Ascorbic acid was extracted and quantified using liquid chromatography according to previous method (Chebrolu, Jayaprakasha, Yoo, Jifon, & Patil, 2012). Each sample was analyzed three times and the ascorbic acid contents were expressed as mg 100 ml^−1^ juice.

### 2.7 Carotenoids analysis

Extraction of carotenoids was performed according to a previously published method with slight modifications (Chaudhary, Jayaprakasha, Porat, & Patil, 2012). Juice samples (10 g) were extracted using chloroform (15 ml) containing BHT (0.2%). An Agilent HPLC 1200 Series (Foster City, CA, USA) system consisting of a solvent degasser, quaternary pump, autosampler, column, oven, and diode array detector was used for quantification. A C‐18, Gemini 5 μm column (250 mm × 4.6 mm i.d.) with a guard cartridge was used (Phenomenex, Torrance, CA, USA). Elution was carried out using a gradient mobile phase of acetonitrile (A) and isopropyl alcohol (B). Carotenoids were detected at 450 nm and quantified using external standard calibration.

### 2.8 Quantification of limonoids, flavonoids, and furocoumarins

#### 2.8.1 Sample preparation

Extraction was carried out according to previously published method with slight modification (Chaudhary et al., 2012). Each juice sample (10 g) was extracted using 15 ml of ethyl acetate on a shaker for 3 hr. The organic layer was separated and the residue was extracted twice. All extracts were pooled and the solvent was evaporated to dryness. The dried residue was reconstituted with 4‐ml acetone, filtered using a 0.45‐μm PTFE filter, and further analyzed for limonoids, flavonoids, and furocoumarins using HPLC.

#### 2.8.2 Quantification of limonoids and flavonoids using HPLC

Limonoids and flavonoids were quantified simultaneously using Waters HPLC (Milford, MA, USA) using spectra model with a PDA detector (2996) coupled with a binary HPLC pump 1525 and 717 plus auto sampler. The chromatographic separations were conducted on a C‐18, Gemini 5 μm column (250 mm × 4.6 mm i.d.) from Phenomenex (Torrance, CA, USA). Limonoids were detected at 210 nm and flavonoids were detected at 280 nm. The entire chromatographic separation was performed with a gradient mobile phase of 0.03 mol/L phosphoric acid (A) and acetonitrile (B) with 1 ml min^−1^ flow rate. Each sample was injected three times. Results were expressed as mg 100 g^−1^ fresh weight.

#### 2.8.3 Quantification of furocoumarins using HPLC

Furocoumarins were analyzed using our previously described method (Chaudhary et al., 2012). Each sample was analyzed in triplicate and the results were expressed as μg 100 g^−1^ fresh weight.

### 2.9 Determination of total phenolics and radical scavenging activity

#### 2.9.1 Sample preparation

Juice samples (10 g) were extracted twice with 20‐ml methanol on a shaker for 3 hr. The extracts from each sample were pooled, filtered using Whatman grade 1 filter paper, and further used for quantification of total phenolics and radical scavenging activity using a DPPH assay. Volume of the extracts was measured for calculating dilution factor.

#### 2.9.2 Total phenolics

The total phenolics contents of methanol extracts were determined using our previously published method and the results were expressed as catechin equivalents (Chaudhary et al., 2012).

#### 2.9.3 Radical scavenging activity

Radical scavenging activity of Rio Red grapefruit methanol extracts was measured according to our previously published method, using the DPPH assay (Chaudhary et al., 2012). Radical scavenging activity was expressed as mg of ascorbic acid equivalent per g of fresh sample weight.

### 2.10 Statistical analysis

One‐way analysis of variance (ANOVA) was performed using PASW Statistics 18 software (SPSS Inc.). A general linear model was used to test significant differences and means were compared using Tukey's HSD test at 5% probability level. The results were expressed as means ± SE.

## 3 Results and Discussion

### 3.1 Total soluble solids and total acidity

In this study, we observed no significant effect of storage temperature on total soluble solids (Table 1), which remained constant in all three treatments throughout the storage period. We observed a slight decrease in total acidity in all three treatments with increasing storage period. Total acidity at the beginning of storage was 1.03%, which decreased to 0.92% after 12 weeks of storage. Consequently, ripening ratio in all treatments increased with storage, due to the slight decrease in acidity (Table 1). A decrease in total acidity during storage is commonly observed and is attributed to consumption of organic acids for energy production (Cohen, Shalom, & Rosenberger, 1990). Previous studies showed similar results, where temperature and storage did not affect TSS (sugar), but reduced the acidity (Porat et al., 2000). The sugar‐to‐acidity ratio (ripening index) is one of the most important factors influencing the taste, and determining the harvest time of the fruits.

**Table 1:** TSS, total acidity, and ripening ratio of Rio Red grapefruit stored for 0, 3, 6, 9, and 12 weeks at 11°C, 5°C, or conditioned (CD)

	Storage duration (weeks)				
	0	3	6	9	12
TSS °Brix					
11°C	11.70 ± 0.11a	11.20 ± 0.22a	11.27 ± 0.27a	11.23 ± 0.23a	11.30 ± 0.20a
5°C	11.70 ± 0.11a	11.36 ± 0.24a	11.23 ± 0.27a	11.37 ± 0.23a	11.67 ± 0.20a
CD	11.70 ± 0.11a	11.73 ± 0.22a	11.63 ± 0.27a	11.60 ± 0.23a	11.97 ± 0.20a
Total acidity (%)					
11°C	1.03 ± 0.02a	0.96 ± 0.05a	0.96 ± 0.05a	0.95 ± 0.05a	0.85 ± 0.05a
5°C	1.03 ± 0.02a	1.06 ± 0.06a	1.00 ± 0.05a	1.01 ± 0.05a	0.92 ± 0.05a
CD	1.03 ± 0.02a	1.02 ± 0.05a	0.99 ± 0.05a	0.99 ± 0.06a	0.92 ± 0.05a
Ripening ratio					
11°C	11.4 ± 0.32a	11.68 ± 0.36a	11.76 ± 0.39a	11.87 ± 0.38a	13.51 ± 0.61a
5°C	11.4 ± 0.32a	10.89 ± 0.40a	11.34 ± 0.39a	11.39 ± 0.38a	12.89 ± 0.61a
CD	11.4 ± 0.32a	11.65 ± 0.36a	11.79 ± 0.39a	11.81 ± 0.46a	13.09 ± 0.61a

TSS, Total soluble solids

Data represent means ± S.E. of three replications, each replication containing two samples prepared from three individual fruits (*n* = 6). Means with different letters indicate significant differences between treatments for each time period (*p *<* *.05).

### 3.2 Incidence of chilling injury

**Figure 1:**
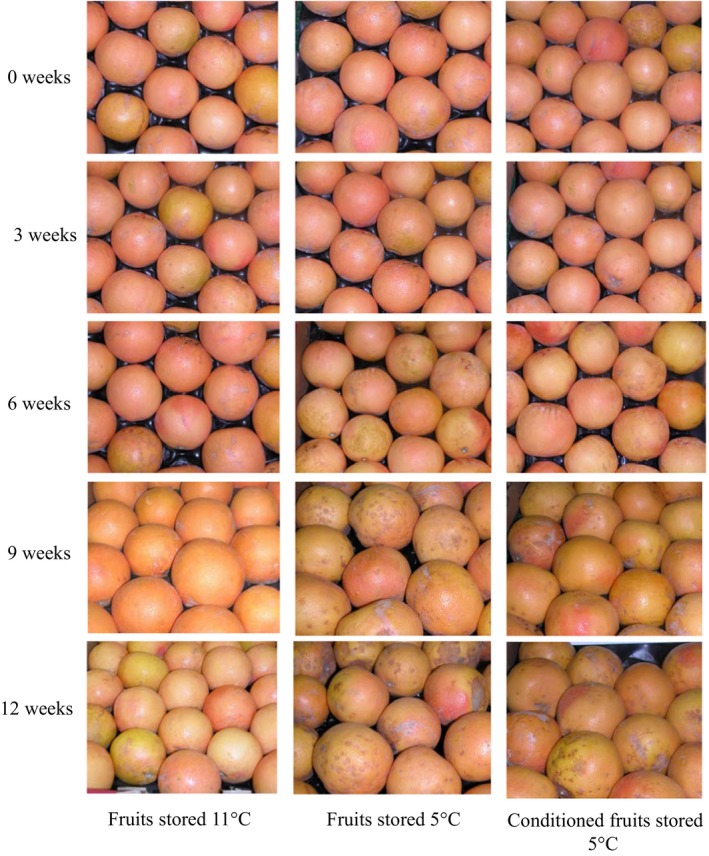
External appearance of ‘Rio Red’ grapefruits observed at 0, 3, 6, 9, and 12 weeks of storage at 11°C, 5°C, or conditioned fruits stored at 5°C

**Figure 2:**
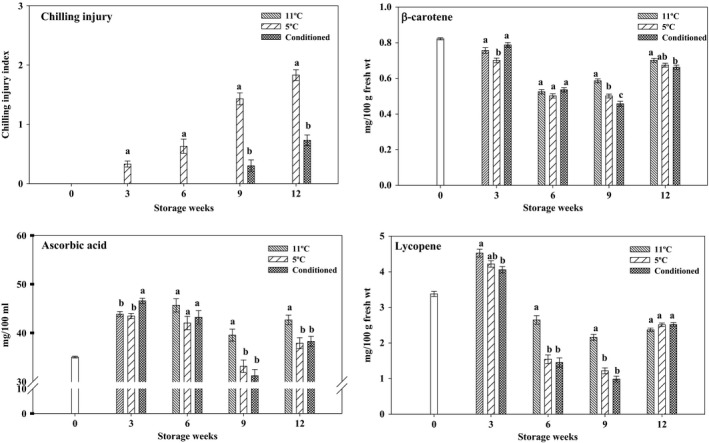
Chilling injury index and levels of ascorbic acid, β‐carotene, and lycopene in pulp of ‘Rio Red’ grapefruit stored at 11°C, 5°C, and in conditioned (CD) fruits. Data represent means ± S.E. of three replications, each replication containing three samples (*n* = 30 for CI index study, each replication containing 10 fruits). Means with different letters indicate significant differences between treatments at each time period (*p *<* *.05)

Grapefruit develops CI, which manifests as pitting or brown staining of the flavedo when stored below 10°C (Figure 1). In this study, no CI symptoms were seen in fruits stored at 11°C throughout the storage period. However, nonconditioned fruits stored at 5°C had severe CI with CI indices of 0.33, 0.63, 1.43, and 1.83 at 3, 6, 9, and 12 weeks of storage, respectively (Figure 2). Conditioning grapefruits at 16°C for 7 days prior to storage significantly reduced and delayed the incidence of CI. Conditioned fruits (CD) showed no CI symptoms until 9 weeks of storage and had significantly lower CI indices (0.30 and 0.73 at 9 and 12 weeks of storage, respectively) as compared to nonconditioned fruits stored at 5°C. CD fruits had CI indices nearly 4.5‐ and 2.5‐fold lower than those of nonconditioned fruits stored at 5°C at 9 and 12 weeks of storage, respectively. Our results agree with previous studies where temperature conditioning delayed and reduced CI in Star Ruby grapefruit (Biolatto, Vazquez, Sancho, Carduza, & Pensel, 2005; Chaudhary, Jayaprakasha, Porat, & Patil, 2014). In conclusion, conditioned fruits can be stored up to 9 weeks with minimal incidence of CI.

### 3.3 Ascorbic acid

Regulating temperature during postharvest operations is one of the most important factor in maintaining fruit quality and extending the shelf‐life. Ascorbic acid is significantly influenced by storage temperature in fruits and vegetables. In this study we investigated the effect of different storage temperatures and conditioning treatment on grapefruit ascorbic acid. Ascorbic acid levels increased during cold storage (5°C and CD) at 3 weeks of storage and then gradually decreased to initial levels at 9 weeks of storage (Figure 2). Ascorbic acid levels in the fruits stored at 11°C gradually increased up to 6 weeks, decreased at 9 weeks, and increased again at 12 weeks of storage. CD fruits had higher ascorbic acid levels at 3 weeks of storage; however, fruits stored at 11°C had significantly higher levels at 9 and 12 weeks of storage. No difference was observed between the two cold storage treatments at 6, 9, and 12 weeks of storage. Ascorbic acid is one of the most important antioxidants in plants, acting to counter biotic and abiotic stress by detoxifying reactive oxygen species produced under stress, with the help of the ascorbate–glutathione cycle (Foyer & Noctor, 2011). In citrus fruits, ascorbic acid usually degrades with increasing storage temperature and period (Adisa, 1986). Chilling injury causes accelerated loss of ascorbic acid in susceptible crops (Lee & Kader, 2000). Besides, CI leads to cellular and oxidative stress, which can affect the levels of ascorbic acid (Stevens et al., 2008). Ascorbic acid levels decreased in cucumbers with CI (Hariyadi & Parkin, 1991). In this study, after 9 weeks, CI incidence increased in both cold storage treatments, which could have led to the decrease in ascorbic acid contents. In addition, other studies also reported that cold storage decreased the ascorbic acid contents in citrus fruits (Pérez, Luaces, Oliva, Ríos, & Sanz, 2005; Rapisarda, Bianco, Pannuzzo, & Timpanaro, 2008). In a previous study, ascorbic acid levels in Star Ruby grapefruit decreased with increase in storage period (Chaudhary et al., 2014).

### 3.4 Carotenoids analysis

Carotenoids in citrus are influenced by storage temperature and are differentially regulated in different tissues (Tao, Wang, Xu, & Cheng, 2012). In this study β‐carotene and lycopene were quantified in juice vesicles during cold storage period (Figure 2). β‐carotene decreased in all three treatments up to 9 weeks of storage and increased at 12 weeks of storage. At 9 weeks of storage, fruits stored at 11°C had significantly higher β‐carotene, followed by fruits stored at 5°C and CD fruits. We observed a small increase in lycopene levels in all treatments at 3 weeks of storage; however, after 3 weeks, the levels gradually decreased up to 9 weeks of storage. Lycopene levels were significantly higher in fruits stored at 11°C at 6 and 9 weeks of storage, with no significant difference observed in fruits stored at cold temperature. Nevertheless, we observed no significant difference in lycopene levels between treatments after 12 weeks of storage. Carotenoid biosynthesis in citrus fruits is temperature dependent, with temperatures of 15–25°C allowing the most carotenoid production (Wheaton & Stewart, 1973). Storage temperatures below 5°C affect carotenoid biosynthesis and cause carotenoid degradation (Tao et al., 2012; Van Wyk, Huysamer, & Barry, 2009). In this study, decrease in carotenoids, mainly lycopene, was more in fruits stored at low temperatures. However, the effect of storage temperature on carotenoid biosynthesis is more prominent in citrus peel than in the pulp (Rodrigo, Alquézar, Alós, Lado, & Zacarías, 2013; Tao et al., 2012). In contrast to our results, in Cara Cara navel orange fruits stored at 4°C for 35 days, the total carotenoid contents increased, including lycopene, in the pulp as compared with the fruits stored at 20°C (Tao et al., 2012). Also, no effect of storage temperature was observed on the carotenoids in the pulp of Satsuma mandarin (Matsumoto, Ikoma, Kato, Nakajima, & Hasegawa, 2009).

### 3.5 Limonoids quantification

**Figure 3:**
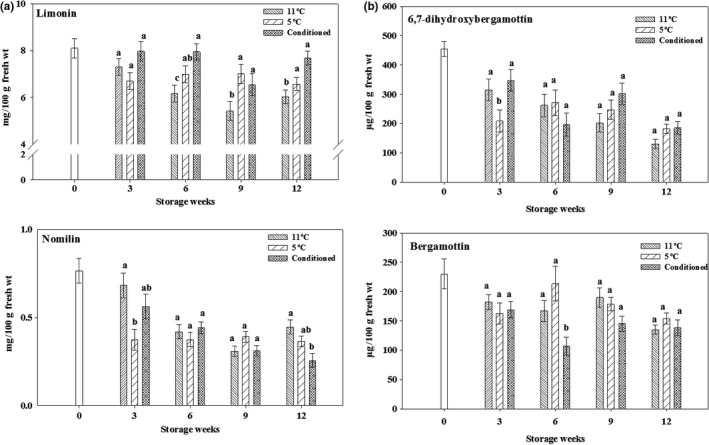
Variation in limonoid (a) and furocoumarin (b) contents of ‘Rio Red’ grapefruit. Fruits were stored for 0, 3, 6, 9, and 12 weeks at 11°C, 5°C, or conditioned (CD). Data represent means ± S.E. of three replications, each replication containing three samples. Means with different letters indicate significant differences between treatments at each time period (*p *<* *.05)

Limonin and nomilin were quantified in this study (Figure 3a). Limonin levels were higher in CD fruits at 6 and 12 weeks, followed by fruits stored at 5°C and 11°C. At 3 weeks of storage, no significant differences were observed in limonin content among the three treatments. Limonin levels were overall maintained in conditioned fruits during the storage period, but they decreased in other two treatments. Nomilin levels decreased in all treatments during the storage period. No significant differences were observed in nomilin levels among the treatments at 6 and 9 weeks of storage. Fruits stored at 11°C had higher nomilin content at 3 and 12 weeks of storage as compared to other two treatments. Limonin and nomilin are limonoid aglycones that have tissue‐specific synthesis and accumulation (Li et al., 2014). Nomilin is also the precursor of limonin in the limonoid biosynthetic pathway, which can affect the levels of limonoids during storage. As fruit matures, limonoid aglycones are converted into nonbitter limonoid glucosides by limonoid glucosyltransferase (Hasegawa et al., 1991).

### 3.6 Furocoumarins quantification

Furocoumarins, namely 6,7‐dihydroxybergamottin (DHB) and bergamottin, were quantified in this study (Figure 3b). Levels of both furocoumarins decreased in all treatments at 12 weeks of storage as compared to the initial storage period. We observed no significant differences in DHB levels among the treatments at 6, 9, and 12 weeks of storage. However, bergamottin levels did not significantly differ among the treatments at 3, 9, and 12 weeks of storage. Furocoumarins are synthesized in response to stress and are linked to other secondary metabolites such as flavonoids and lignins through L‐phenylalanine *via* the shikimate pathway. In addition, several pre‐ and postharvest factors influence the levels of furocoumarins in grapefruit (Chaudhary et al., 2012; Girennavar, Jayaprakasha, & Patil, 2008). As compared to DHB, bergamottin is more stable in grapefruit (Girennavar et al., 2008) which was also observed in this study, where bergamottin degraded relatively less than DHB in all treatments. White flesh grapefruits have higher furocoumarins than red varieties (Girennavar et al., 2008). Both DHB and bergamottin can strongly inhibit CYP3A4 enzymes, which cause drug interactions, with DHB being more potent than bergamottin (Girennavar, Poulose, Jayaprakasha, Bhat, & Patil, 2006).

### 3.7 Flavonoids quantification

In this study, five flavonoids, namely, narirutin, naringin, neohesperidin, didymin, and poncirin were quantified (Fig. S1). Naringin is the main bitter flavonoid present in grapefruit juice, along with poncirin and neohesperidin. Naringin and narirutin were the major flavonoids detected. Narirutin, naringin, neohesperidin, and poncirin levels did not differ significantly among the treatments at 6 and 9 weeks of storage (Table 2). Naringin, neohesperidin, and didymin were lower in fruits stored at 5°C at 3 and 12 weeks of storage, whereas CD fruits showed significantly higher levels followed by fruits stored at 11°C. Overall, flavonoids were at similar or at higher levels in CD fruits as compared to other two treatments. Storage period had significant effect on narirutin, naringin, neohesperidin, and poncirin. Flavonoid content was significantly higher (*p *<* *.05) in most treatments at 6 weeks and lower at 9 weeks after storage. The higher levels may be attributed to increase in stress during prolonged storage for fruits stored at 11°C, while increase in chilling stress for other two cold storage treatments. Change in temperature after conditioning treatment (16°C) to cold storage (5°C) may have influenced flavonoid biosynthesis leading to higher levels of naringin and poncirin at 3 weeks in conditioned fruits. Lower levels of flavonoids were observed at 9 weeks in both cold treatments, especially in CD fruits, when chilling injury incidence increased. Flavonoids and phenolic acid play important role in modulating various abiotic and biotic stresses in plants and fruits. Previous studies have reported induction of the enzymes involved in the phenylpropanoid pathway, especially phenylalanine ammonia lyase under stress conditions (Crifò, Puglisi, Recupero, & Lo Piero, 2011; Lafuente, Zacarias, Martínez‐Téllez, Sanchez‐Ballesta, & Granell, 2003). Flavonoids are polyphenols biosynthesized from the phenylpropanoid pathway and have been reported to be affected by storage temperature (Rapisarda et al., 2008).

**Table 2:** Influence of storage temperature and duration on flavonoid content in juice of ‘Rio Red’ grapefruit. Fruits were stored for 0, 3, 6, 9, and 12 weeks at 11 or 5°C or conditioned (CD)

	Weeks of storage				
	0	3	6	9	12
Narirutin					
11°C	13.88 ± 0.37a	14.60 ± 0.41a	15.28 ± 0.54a	14.28 ± 0.82a	16.06 ± 0.49a
5°C	13.88 ± 0.37a	13.28 ± 0.41b	15.69 ± 0.55a	14.06 ± 0.82a	14.70 ± 0.46a
CD	13.88 ± 0.37a	15.14 ± 0.50a	15.84 ± 0.54a	14.72 ± 0.92a	15.29 ± 0.48a
Naringin					
11°C	68.28 ± 1.90a	77.54 ± 1.82b	80.38 ± 2.39a	70.29 ± 4.43a	76.72 ± 2.13a
5°C	68.28 ± 1.90a	70.98 ± 1.82c	76.69 ± 2.39a	65.36 ± 4.27a	68.73 ± 1.89b
CD	68.28 ± 1.90a	85.31 ± 2.04a	75.70 ± 2.24a	69.42 ± 5.05a	76.25 ± 2.13a
Neohesperidin					
11°C	2.33 ± 0.09a	2.37 ± 0.08ab	2.73 ± 0.08a	2.50 ± 0.16a	2.70 ± 0.07a
5°C	2.33 ± 0.09a	2.11 ± 0.08b	2.51 ± 0.08a	2.34 ± 0.16a	2.40 ± 0.06b
CD	2.33 ± 0.09a	2.44 ± 0.09a	2.66 ± 0.07a	2.37 ± 0.17a	2.51 ± 0.06ab
Didymin					
11°C	2.49 ± 0.10a	2.42 ± 0.08b	2.36 ± 0.10b	2.34 ± 0.23a	2.59 ± 0.08a
5°C	2.49 ± 0.10a	2.17 ± 0.08c	2.50 ± 0.10ab	2.62 ± 0.23a	2.33 ± 0.07b
CD	2.49 ± 0.09a	2.73 ± 0.09a	2.74 ± 0.10a	2.41 ± 0.25a	2.62 ± 0.07a
Poncirin					
11°C	9.42 ± 0.30a	10.62 ± 0.38a	10.35 ± 0.52a	9.24 ± 0.42a	9.32 ± 0.43a
5°C	9.42 ± 0.30a	9.46 ± 0.38b	9.84 ± 0.52a	8.23 ± 0.42a	9.13 ± 0.41a
CD	9.42 ± 0.30a	12.03 ± 0.46a	10.36 ± 0.52a	9.31 ± 0.47a	10.34 ± 0.42a

Results expressed as mg 100^−1^ g fresh weight. Data represent means ± S.E. of three replications, each replication containing three samples. Means with different letter indicate significant differences between treatments at each time period (*p *<* *.05).

### 3.8 Total phenolics

**Figure 4:**
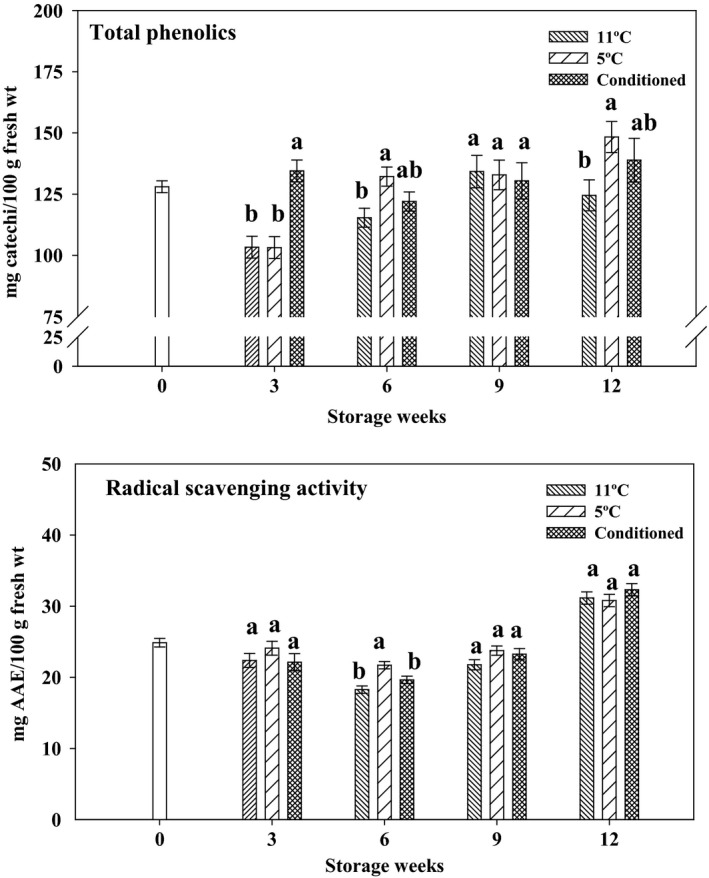
Total phenolics and radical scavenging activity of methanol extracts of Rio Red grapefruits stored for 0, 3, 6, 9, and 12 weeks at 11°C, 5°C, and in conditioned fruits stored at 5°C. Data represent means ± S.E. of three replications, each replication containing three samples. Means with different letters indicate significant differences between treatments at each time period (*p *<* *.05)

Total phenolics were significantly higher in CD fruits at 3 weeks of storage as compared to other treatments (Figure 4). However, at 6 and 12 weeks of storage, fruits stored at 5°C had higher phenolics followed by conditioned fruits. This could be due to an increase in CI in fruits stored at cold temperatures. Phenolic compounds in grapefruit mainly comprise flavanone glucosides, *p*‐hydroxybenzoic acids, and hydroxycinnamic acids, which are influenced by different abiotic and biotic stresses (Chebrolu, Jayaprakasha, Jifon, & Patil, 2012). The fruits stored at 5°C had higher CI, followed by CD fruits, and total phenolics showed a similar trend. Our previous study in Star Ruby grapefruit also showed an increase in total phenolics during storage (Chaudhary et al., 2012).

### 3.9 Radical scavenging activity

Radical scavenging activity was measured using a DPPH assay (Figure 4). Fruits stored at 5°C had higher radical scavenging activity at 6 weeks of storage. However, we observed no significant differences among the different treatments at 3, 9, and 12 weeks of storage. Antioxidant activity of all three treatments increased at 12 weeks of storage. Ascorbic acid and phenolics are the main contributors to antioxidant activity in citrus fruits, with ascorbic acid contributing more than 65% of total antioxidant activity (Gardner, White, McPhail, & Duthie, 2000). In addition, a synergistic effect of phenolics and ascorbic acid can influence antioxidant activity (Huang, Ou, & Prior, 2005).

## 4 Conclusion

Low‐temperature conditioning treatment can effectively reduce the incidence of CI. Overall, conditioning treatment did not affect most of the health‐promoting compounds present at the end of 12 weeks of storage. Fruits stored at 5°C without conditioning had lower health promoting compounds at 3 weeks of storage. However, at 12 weeks of storage, all three treatments showed similar levels of lycopene, narirutin, poncirin, and furocoumarins. Generally, conditioning treatment maintained the levels of most of the health‐promoting compounds assessed as compared to nonconditioned fruits stored at 5°C. Storage period significantly affected all health‐promoting compounds. Low‐temperature conditioning of the fruits prior to quarantine treatments or cold storage is recommended to prevent CI and to maintain health‐promoting compounds.

## Conflict of Interest

None declared.
