# Supplementary material for: Influence of storage temperature and low‐temperature conditioning on the levels of health‐promoting compounds in Rio Red grapefruit
Source: Food Sci Nutr. 2016 Oct 2;5(3):545–53. doi: 10.1002/fsn3.429 (PMC5448389; doi:10.1002/fsn3.429)
Supplement: Supplementary file 1 [file FSN3-5-545-s001.docx]

**Supplementary Figure 1.** (A) Structures of flavonoids detected in grapefruit juice. (B) HPLC chromatograms of limonoids and flavonoids analyzed at 210 and 280 nm respectively. Peak 1– narirutin, 2-naringin, 3- neohesperidin, 4- didymin, 5- poncirin, 6- limonin, and 7- nomilin.
